# Editorial: Green lifestyle transformation in the digital era

**DOI:** 10.3389/fpsyg.2025.1576604

**Published:** 2025-03-17

**Authors:** Shengxiang She

**Affiliations:** ^1^Business School, Guangzhou College of Technology and Business, Guangzhou, China; ^2^Institute of Science Innovation and Culture, Rajamangala University of Technology Krungthep, Bangkok, Thailand

**Keywords:** climate change, green lifestyle, digital platform, environmental sustainability, pro-environmental behaviors

## 1 Introduction

The urgency of climate action, underscored by the Climate Change 2023: A Synthesis Report released on March 20, 2023, by the Intergovernmental Panel on Climate Change (IPCC), demands a radical reimagining of how societies consume, interact, and sustain themselves. As global warming threatens to breach the 1.5°C threshold, the digital era presents a paradox: while technology accelerates resource-intensive lifestyles, it also holds unprecedented potential to catalyze green transformations. This Research Topic, Green Lifestyle Transformation in the Digital Era, explores how digital tools—from social media to serious games—can reshape behaviors, bridge intention-action gaps, and embed sustainability into the fabric of daily life. By integrating psychological, cultural, and systemic perspectives, the contributions illuminate pathways to leverage technology for equitable, scalable, and enduring environmental change.

This Research Topic seeks to decode the psychological and sociocultural mechanisms driving sustainable behaviors in digital contexts, evaluate the role of institutions, technologies, and policies in scaling green practices, and address gaps between individual actions, systemic incentives, and planetary outcomes.

After a meticulous and rigorous review process, we have selected seven papers that not only align seamlessly with our theme but also exhibit exceptional research contributions. Regrettably, while numerous other submissions held merit, they did not meet our criteria for selection for various reasons. Our Research Topic attracted a total of 37 submissions, of which only seven were accepted, yielding an acceptance rate of 18.9%. These accepted papers, authored by 17 esteemed scholars, collectively emphasize that digitalization transcends its role as a mere tool; it is a transformative force that must be ethically navigated to harmonize human behavior with ecological boundaries.

## 2 Cultural and spiritual foundations of sustainability

Liu et al. demonstrate how spiritual values can drive tangible environmental action. By examining Wat Chak Daeng's plastic waste management—rooted in Buddhist principles of reducing, reusing, and recycling—the study challenges technocentric solutions. It reveals how cultural narratives, when amplified through community engagement, foster intrinsic motivation and scalable practices. This work underscores the need to integrate local wisdom into global sustainability frameworks.

## 3 Education and institutional catalysts

Chen C. et al.'s highlights universities as incubators of sustainability. Analyzing Chinese students, the study shows how green campus initiatives, institutional policies, and hands-on education cultivate eco-conscious intentions and habits. The findings advocate for holistic institutional ecosystems that normalize sustainability, from cafeterias to curricula, shaping future generations' lifestyles.

## 4 Behavioral spillovers and intergenerational dynamics

Behavioral spillovers emerge as critical levers. Wang and Zhang reveals how children's actions trigger parental guilt, motivating families to adopt greener practices. Conversely, Frezza's applies the Identity and Practice Interdependence Framework to Brazilian steelworkers, showing how workplace sustainability programs—when reinforced by socio-material support—spark home routines. Both studies emphasize the ripple effects of structured interventions across life domains.

## 5 Digital tools as behavioral architects

The digital realm's power to shape choices is dissected across three articles. Chen J. et al.'s proposes an Embodied-Enactive Cognition Model, arguing that immersive gameplay fosters pro-environmental decision-making by merging cognitive, emotional, and sensory engagement. Meanwhile, Wu and Long's links social media marketing to green consumerism, identifying trust and perceived value as key mediators. Finally, Chen X. et al.'s demonstrates how vivid digital narratives evoke eco-guilt and empathy, steering tourists toward sustainable choices. Together, these articles map how digital platforms—whether gamified, social, or immersive—can turn abstract climate goals into relatable, actionable behaviors.

## 6 Broader context and imperatives

The contributions collectively reframe sustainability as a sociotechnical challenge. Digital tools alone cannot drive transformation; they must intersect with cultural values, institutional accountability, and policy frameworks. For instance, while Ant Forest-style gamification engages millions, its impact depends on equitable access to technology and safeguards against greenwashing. Similarly, workplace spillovers require corporate policies that reward sustainable identities, not just compliance.

The Research Topic also exposes critical tensions: Can digital platforms reconcile individualized nudges with collective systemic change? How do we balance data-driven efficiency with privacy and inclusivity? These questions demand interdisciplinary collaboration, bridging environmental science, behavioral psychology, and tech ethics.

## 7 Conclusion

Green Lifestyle Transformation in the Digital Era is a call to reorient technology's role—from enabling convenience to fostering stewardship. The articles illustrate that sustainability is not a solitary pursuit but a networked endeavor, shaped by families, schools, workplaces, and algorithms. As editors, we envision this Topic as a catalyst for designing digital ecosystems that amplify empathy, equity, and ecological resilience. The path forward lies not in techno-utopianism but in grounding innovation in humanity's deepest values—ensuring bytes serve both people and the planet.

